# Ten Letters of Interest with Replies

**Published:** 1875-10

**Authors:** 


### Our Correspondence.

PAW PAW, MICH., July 1st, '75.

DEAR BISTOURY:

I had concluded not to take the BISTOURY another year, on account of my age and poor eyes—now over 77 years of age; but the BISTOURY is so very good—let it come.

Yours, Truly,

J. A. SHELDON, M. D.

---

WAVERLY, N. Y., Aug. 17.

EDITOR BISTOURY:

Will you please inform me through the BISTOURY, of a medicine that will do away with worms from persons. Y.

You will find remedies mentioned in the following numbers of the BISTOURY: Vol. V, No. 2, Page 49—Vol. VI, No. 2, Page 147, also in Medical Items and News, present number.

---

HANNIBAL, MO., July 25th, 1875.

DR. UP DE GRAFF:

I can sympathise with your correspondent in last number of BISTOURY, who consumed those six bottles of patent medicine without relief. I tried the same thing myself, but stopped off short after your advice to him. Did he follow your directions and take the medicines prescribed? If so, am trembling to hear the result. M.

We fear he has, for we have not heard from him since.

---

OWEGO, N. Y. June 16, 1875.

DEAR DOCTOR:

We have a son, twelve years old, who injured his eye, four years ago, by exploding percussion caps. The doctor who attended him thought no portion of the metal had lodged in the eye, but that the painfulness of the organ was caused solely by the cut upon the cornea. His eye healed up in two or three months, but every now and then it has spells of paining him, when his well eye bothers him precisely as described in the July number of the BISTOURY, in the article upon accidents to the eye. Since reading that article we have thought there might be something in his eye, and if there really is, do you advise us to have that eye removed? We have become very anxious about it lately because of trouble it causes the well eye. J. B.

What was written in the article referred to, applies in this case. If the diseased eye is painful and irritable at times, causing sympathetic inflammation in the opposite one, the offending eye should be at once removed. It is of no use when blind and shrunken away, and must endanger the seeing one to the loss of vision. Our advice is to have it extirpated.

---

PATERSON, N. J., Sept. 6th, 1875.

EDITOR BISTOURY:

I have a son, just ready to enter College, aged nineteen, who has a queer anomoly of vision, and which annoys him very much. He can see the rails in a fence distinctly; but cannot see the pickets. So with objects lying upon the ground, he can see them better than when held erect. That is to say, he can see vertical lines, but not horizontal ones. What is the difficulty, and can it be corrected? L. B. S.

Your son has *astigmatism*, described in the April number, 1872, of the BISTOURY. Cylindrical lenses, which can only be prescribed by an oculist, will correct the difficulty.

---

WHEELING, W. VA., Sept. 8th, 1875.

DR. UP DE GRAFF:

Some time ago, I saw a very interesting article in the BISTOURY, upon deformed toes. This induces me to write you to learn whether you know of any means for removing corns. I have a very troublesome one or my little toe,—just on top,—and have tried everything under the sun that I ever heard of that was good for corns. I bought a bottle of Johnson's corn remedy, which blistered my toe and laid me up for a week. Then I tried angle-worm oil, salt pork, nitric acid, chloride of zinc, &c., &c. The last two remedies nearly killed me, and swelled my toe up larger than the great toe, and caused bunches like warts to grow all over it. I can't wear a shoe, nor can I get out doors. What *can* a fellow do in such a predicament? R.

Procure a chisel—a sharp one; place your toe,—the tender and deformed one,—upon a solid block, allowing your foot to rest there likewise. Then, place the chisel, with the sharp edge resting upon the toe, close to where it joins the foot. Next, call in your big brother,—or some other fellow's brother will do as well, arm him with a blacksmith's sledge, and direct him to strike upon the upper or wooden end of that chisel, with all his might. Have him exert due caution, in order that the hammer may hit the chisel and not your foot. Finally, send for a surgeon—he may be needed.

HEALTH DEPARTMENT,
OFFICE OF HEALTH OFFICER.
MILWAUKEE, July, 30 1875.

EDITOR BISTOURY:

Enclosed you will find 50 cents for the BISTOURY. It is the most honest value for the money, to be found in the periodical literature of the country. To persons outside of the profession, your Quarterly Journal contains very useful information, communicated in a very pleasing manner.

Respectfully yours,

JAMES JOHNSON, M. D.,
Health Officer.

Thanks, doctor; we are delighted to receive the sanction and encouragement of our professional brethren.

---

AUBURN, N. Y., July 20, 1875.

DR. UP DE GRAFF:

Dr. ——, who comes to our town occasionally, operated upon our little son for cross-eye. We paid him his price, and he assured us the eye would come all straight, but it don't. Since then the Doctor has not been here, and we do not know where to find him. What shall we do about it? Will it answer to let it go until he *does* come?

MRS. J. R.

Of course it will, because then you will be perfectly safe and no further injury can come to your child's eye, for it is not at all probable that you will see your traveling doctor again. Therefore, wait for him, but in the meantime, it would be well to consult some reliable surgeon—who remains at home for patronage—and have the child's eye properly attended to.

---

MINNEAPOLIS, MINN., Sept. 9th, 1875.

EDITOR BISTOURY:

We have a little girl, aged ten, who has very red and irritable looking eye-lids. The margins of the lids are sore, and among the eye-lashes is collected a hard, scaly matter, which, when removed, leaves the margin sore and bleeding. Lately, her lashes begin to fall out, giving the lid a naked look. We would bring her to you for advice or treatment, were we not so far away. Cannot you prescribe for us without our coming east?

S. K. M.

The child is suffering from a disease of the *meibomian* glands, which deposit an oily secretion at the margins of the eye-lids, but when inflamed throw out matter, which becomes encrusted among the lashes, and inflames the entire lid.

Have your druggist prepare for you the following salve, which rub into the roots of the lashes morning and evening, first bathing the lids in warm water and removing all the secretion. By continuing this application for three or four months you will completely remove the disease:—

℞

Red oxide of mercury, gr. xv.;
Simple cerate, ℥j. Mix thoroughly.

---

Here follows a racy letter from a subscriber in Texas. He encloses an account of a fishing excursion in that far-off country, but is too long to print in the BISTOURY. The doctor writes a good letter, and we're sure our readers will be glad to see him in the BISTOURY again. Come again, doctor.

NAVASOTA, TEXAS, Aug. 14, '75.

DR. T. S. UP DE GRAFF:

*Dear Doctor.*—I am the $\frac{1}{10}$ man who did not skip your piece on "Angling," in July No. of the BISTOURY; in fact I skip very little in that journal, and am very careful to read everything from your pen.

In "Angling," you expatiate so extensively on the various pleasures of the spree, that I frequently wished I was there to enjoy some of the fun.

Out here in Texas, on the red waters of the Brazos de Dios, we have not much fun "fyshynge," as the piscatorial tribes generally are large and coarse, such as buffalo, gaspagon, mud cats, loggerhead turtles and alligators.

But, I sat down merely to say a few words and enclose you this "companion piece" to yours, and I feel satisfied you will thank me for sending it to you. It was copied into the *Wheeling Register* which a friend sent me, who is travelling in that region for health. You see they had the counterpart of your Mrs. Gray, in Mrs. Parsons, and a better part (?) in Miss Wilkinson.

We have had a very dry summer and the crops are cut off in most places, being shorter than was planted for and expected in May and early June. Cotton is being picked rapidly—I never saw the balls open so rapidly as they do now. Where the hands picked a week ago, the fields are quite as white as they were before picking. You are aware the plant resembles most of tropical productions in having blossoms, young fruit, mature fruit (balls) and open balls all at the same time; so there is a constant and long succession of crops till checked by the frost. We cultivate no wheat right here, but grow corn, oats, millet and a little rye and barley.

The season has been no more sickly (excuse the phrase) than usual, but some more fatality attends the fevers. No epidemic. We fear the yellow fever may come from New Orleans, Galveston, Houston, and on up to this point. We had a fear ful yellow fever epidemic here in 1867.

After expressing my high appreciation of the BISTOURY, and wishing it and you long life, I close by subscribing myself,

Very Respectfully,

A. R. KILPATRICK, M. D.

